# Analysis of *SEC9* Suppression Reveals a Relationship of SNARE Function to Cell Physiology

**DOI:** 10.1371/journal.pone.0005449

**Published:** 2009-05-06

**Authors:** Daniel C. Williams, Peter J. Novick

**Affiliations:** Department of Cell Biology, Yale University School of Medicine, New Haven, Connecticut, United States of America; Lawrence Berkeley National Laboratory, United States of America

## Abstract

**Background:**

Growth and division of *Saccharomyces cerevisiae* is dependent on the action of SNARE proteins that are required for membrane fusion. SNAREs are regulated, through a poorly understood mechanism, to ensure membrane fusion at the correct time and place within a cell. Although fusion of secretory vesicles with the plasma membrane is important for yeast cell growth, the relationship between exocytic SNAREs and cell physiology has not been established.

**Methodology/Principal Findings:**

Using genetic analysis, we identified several influences on the function of exocytic SNAREs. Genetic disruption of the V-ATPase, but not vacuolar proteolysis, can suppress two different temperature-sensitive mutations in *SEC9*. Suppression is unlikely due to increased SNARE complex formation because increasing SNARE complex formation, through overexpression of *SRO7*, does not result in suppression. We also observed suppression of *sec9* mutations by growth on alkaline media or on a non-fermentable carbon source, conditions associated with a reduced growth rate of wild-type cells and decreased SNARE complex formation.

**Conclusions/Significance:**

Three main conclusions arise from our results. First, there is a genetic interaction between *SEC9* and the V-ATPase, although it is unlikely that this interaction has functional significance with respect to membrane fusion or SNAREs. Second, Sro7p acts to promote SNARE complex formation. Finally, Sec9p function and SNARE complex formation are tightly coupled to the physiological state of the cell.

## Introduction

Cell growth and division requires the addition of membrane and protein to the surface of the growing cell through the fusion of secretory vesicles with the plasma membrane [1], [2]. The molecules involved in membrane fusion are conserved from yeast to humans, and include the SNARE proteins, defined by a ∼70 amino-acid alpha-helical SNARE motif [3], [4]. The SNARE motif of SNARE proteins on vesicles and on the plasma membrane assemble into a very stable four-helix bundle called the SNARE complex. Although SNARE complex formation is thought to provide the driving force for membrane fusion, accessory proteins influence SNARE assembly and help couple SNARE assembly to fusion and ensure membrane traffic at the correct time and place within a cell.

The yeast exocytic SNAREs consist of the synaptobrevin homologues Snc1/2p on the secretory vesicle and the syntaxin homologues Sso1/2p and SNAP25 homologue Sec9p on the plasma membrane [5]. Analogous to the neuronal SNARE complex, Snc1/2p and Sso1/2p each contribute one helix to the SNARE complex, while Sec9p contributes two helices [6]. *SEC9* is an essential gene originally identified through the isolation of recessive temperature-sensitive alleles, such as *sec9-4*, that cause defects in secretion at the non-permissive temperature [7]. The *sec9-4* mutation encodes a Gly to Asp amino acid substitution in the N-terminal helical domain of Sec9p that reduces the ability of Sec9-4p to complex with Sso1/2p and Snc1/2p [8]. Another temperature-sensitive allele (*sec9-7*) results in a lower cut-off temperature, but does not affect SNARE complex formation *in vitro* [9] suggesting multiple functions for Sec9p. Snc1/2p and Sso1/2p are encoded by redundant yet essential genes: yeast lacking either Snc1p and Snc2p or Sso1p and Sso2p are defective in secretion and accumulate secretory vesicles [10], [11].

SNAREs are thought to constitute the core fusion machinery and considerable work has focused on the identification of additional components that may play a role in membrane fusion. One such component is the vacuolar H+ ATPase (V-ATPase), a multi-subunit complex whose primary function is acidification of intracellular organelles by coupling ATP hydrolysis with translocation of protons across membranes [12]. The V-ATPase is composed of two distinct and separable sectors: the V1 sector is cytosolic and contains the ATPase activity, while the trans-membrane V0 sector forms the proton translocation channel. Three lines of evidence support a role for the V-ATPase in membrane fusion. First, studies of homotypic vacuolar membrane fusion have suggested that the V0 sectors on opposing membranes can form a proteolipid fusion pore and that radial dissociation and expansion of V0 sectors results in membrane fusion [13], [14]. Second, genetic analysis in different model systems has suggested that the V-ATPase can contribute to membrane fusion, independent of vesicle acidification [15]–[17]. Finally, V-ATPase subunits and SNARE proteins have been shown to interact on synaptic vesicles, although the functional significance of this interaction has not been established. [15], [18]

Another possible regulator of SNARE function is Sro7p and its redundant homologue *SRO77*. Sro7p and Sro77p have been implicated in secretion based on genetic studies demonstrating decreased secretion of invertase and an accumulation of secretory vesicles in the absence of *SRO7* and *SRO77* [19]. *SRO7* was initially isolated as a high-copy suppressor of *rho3* mutants, suggesting a role for Sro7p in maintenance of actin polarity [20], [21]. However, further studies have established that the primary role for Sro7p is in membrane fusion. First, Sro7p binds directly to Sec9p, and the interaction between Sro7p and SNAREs is essential for Sro7p function [22], [23]. Second, Sro7p is an effector of the Rab GTPase Sec4p, which has multiple functions during secretion, one of which occurs after vesicle transport to sites of secretion [24]. Finally, tomosyn, which is closely related in sequence with Sro7p, has been implicated directly in vesicle fusion in different systems [25], [26]. While Sro7p is likely to be involved in membrane fusion through an interaction with Sec9p, a role for Sro7p in SNARE complex assembly has not been determined.

Here, we describe genetic and physiological influences on SNARE complex formation. A forward genetic selection was performed to isolate mutations that suppress the temperature-sensitive phenotype of *sec9*-4 mutants. This screen revealed that disruption of the V-ATPase suppresses the temperature-sensitive growth phenotype caused by mutations in *SEC9*. Suppression was not accompanied by increased SNARE complex formation, suggesting that suppression involves a mechanism other than restoration of SNARE complex formation between Sso1/2p, Snc1/2p, and mutant alleles of Sec9p. Improved growth of *sec9* mutants was also observed under conditions in which SNARE complex assembly and the growth rate of wild-type cells was reduced. Thus, suppression is likely the result of lowering the secretory demands of the cell to match the reduced level of Sec9p function. Furthermore, our results suggest that SNARE complex formation is highly responsive to the physiological state of the cell.

## Results

### Disruption of the V-ATPase suppresses *sec9-4*

The *sec9-4* mutation disrupts the first SNARE-forming helix of Sec9p, preventing the formation of dimeric SNARE complexes between Sec9-4p and Sso1/2p and partially inhibiting the formation of trimeric SNAREs [8]. The phenotypic consequences of the *sec9-4* mutation is a temperature-sensitive growth phenotype as *sec9-4* cells display wild-type growth characteristics at 25°C, but are unable to form colonies at 35°C on rich media [7], [8]. We reasoned that mutations that restore growth to *sec9-4* cells at the non-permissive temperature might do so by increasing SNARE complex formation. To isolate such mutations, we performed a forward genetic selection for extragenic suppressors of the *sec9-4* temperature-sensitive phenotype [27]. Suppressors were obtained by plating *sec9-4* cells on rich media and selection of colonies that formed at the non-permissive temperature. These colonies were then screened for secondary phenotypes to facilitate cloning of the suppressor gene. The isolated strains were backcrossed to determine if the suppressor was genetically linked to the secondary phenotype and to separate the suppressor from the *sec9-4* mutation. Two independent suppressing mutations shared the secondary phenotypes of cold-sensitivity and poor growth on glycerol in *SEC9* cells. In both cases the suppressor locus was tightly linked to the secondary growth defects and segregated 2∶2 during tetrad analysis, indicating that they were the result of a single mutation. Furthermore, both the suppression and the secondary phenotypes were found to be genetically recessive to wild type, suggesting that they are the result of a loss-of-function mutation.

Although the phenotypes caused by these two mutations were indistinguishable, they complemented each other indicating that they represent alleles of different loci (data not shown). The wild-type allele of each mutant gene was isolated and identified by complementation cloning: mutants were transformed with a genomic library and colonies able to grow on glycerol were selected. The plasmids conferring rescue were recovered and the genomic fragment contained within the rescuing plasmid was identified by restriction analysis and sequencing. From each strain, the rescuing plasmid contains a genomic fragment encoding a subunit of the V-ATPase (*VMA1 and VMA16*), suggesting that mutations in V-ATPase genes are able to suppress *sec9-4*.

**Figure 1:**
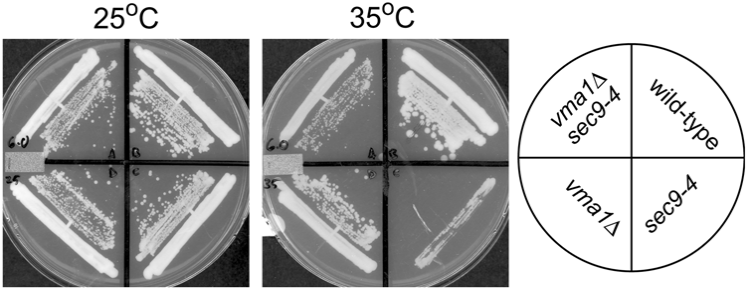
Disruption of the V-ATPase suppresses the *sec9-4* temperature-sensitive phenotype. Isogenic strains of the indicated genotype were plated on YPD media and incubated at 25°C or 35°C for 3 days. At 25°C, cells of all genotypes display growth and are able to form individual colonies. By contrast, at 35°C, *sec9-4* cells grow poorly and do not form individual colonies while *sec9-4 vma1Δ* cells show increased growth and colony formation.

To directly test whether disruption of the V-ATPase is able to suppress mutations in *SEC9*, we generated a deletion allele of the V1 sector *VMA1* and asked whether the deletion allele suppresses *sec9-4* mutations [28]. Trans-heterozygous diploids were sporulated and subject to meiotic analysis and spores from tetra-type tetrads scored for growth at the *sec9-4* permissive and non-permissive temperatures. At the permissive temperature, all four meiotic products displayed growth and were able to form individual colonies (Figure 1). At the non-permissive temperature, *sec9-4* mutant cells grew very poorly and failed to form individual colonies, while *sec9-4 vma1Δ* double mutant cells grew and formed colonies. The suppression is not specific for disruption of the V1 ATPase sector of the V-ATPase, because disruption of *VMA16*, which encodes a subunit of the V0 transmembrane sector [29], is also able to suppress *sec9-4* (data not shown). These results indicate that disruption of the V-ATPase suppresses the temperature-sensitive growth phenotype of *sec9-4*.

### Suppression is not due to defects in vacuolar proteolysis

**Figure 2:**
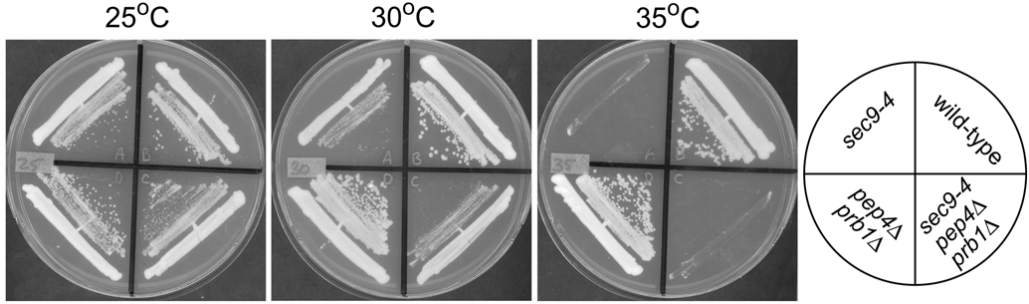
Suppression of *sec9-4* is not due to defects in vacuolar proteolysis. Strains of the indicated genotype were plated on YPD media and incubated at the indicated temperature for 3 days. At all temperatures, the growth characteristics of *sec9-4* cells are identical to *sec9-4 pep4Δ prb1Δ* cells.

The loss of vacuole acidification in V-ATPase mutants prevents zymogen activation and causes defects in the proteolytic function of the vacuole [30], [31]. If the suppression of *sec9-4* by disruption of the V-ATPase is due to defects in vacuolar proteolysis, then mutations that prevent zymogen activation should also suppress. To test this hypothesis, we obtained deletion mutations in *PEP4* and *PRB1*, which are required for the activation of vacuolar proteases, and tested whether these deletions are able to suppress *sec9-4* [32]–[34]. At the *sec9-4* semi- and non-permissive temperatures the growth of *sec9-4* cells was indistinguishable from *sec9-4 pep4Δ* or *sec9-4 prb1Δ* double mutants, indicating that disruption of vacuolar proteolysis is unable to suppress *sec9-4*. In addition, we compared *sec9-4* cells to *sec9-4 pep4Δ prb1Δ* triple mutants (Figure 2) and did not observe any differences in growth or colony formation at any temperature. These results suggest that suppression of *sec9-4* by disruption of the V-ATPase is not due to defects in the proteolytic function of the vacuole.

### Growth under alkaline conditions suppresses *sec9* temperature-sensitive alleles

**Figure 3:**
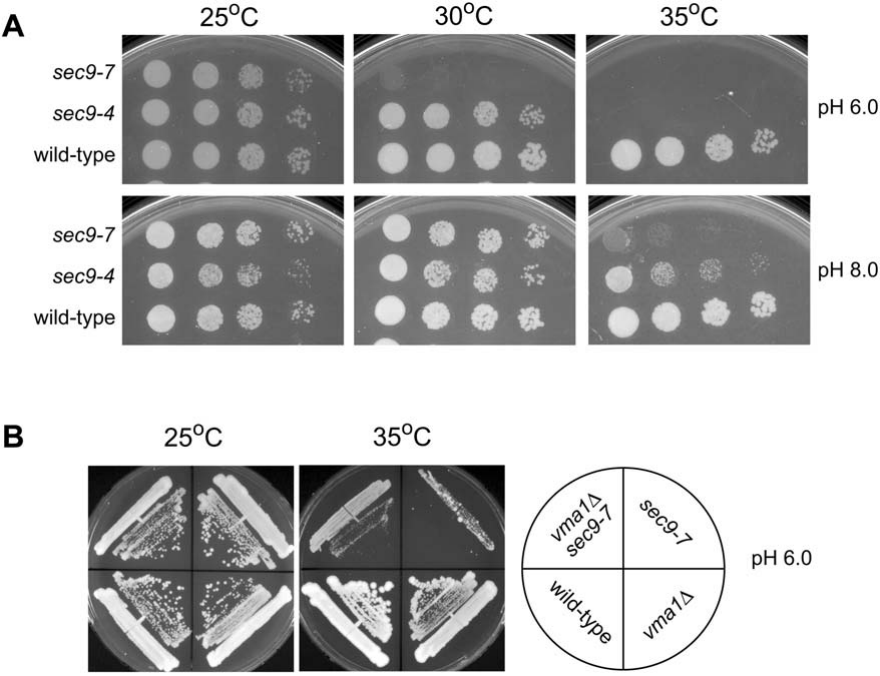
Growth under alkaline conditions suppresses temperature-sensitive alleles of *sec9*. (A) Serial dilutions of *sec9-7*, *sec9-4*, or wild-type cell suspensions were spotted onto YPD media buffered at pH 6.0 or pH 8.0 and incubated at the indicated temperature for 3 days. On pH 6.0 media at 35°C, neither *sec9-7* nor *sec9-4* cells are able to grow and at 30°C *sec9-7* cells grow very poorly, relative to wild type. On media buffered to pH 8.0, both *sec9-4* and *sec9-7* cells show increased growth characteristics at non-permissive temperatures. At 30°C, *sec9-7* cells display wild-type growth, while at 35°C, *sec9-4* cells show increased growth and are able to form individual colonies. (B) Strains of the indicated genotype were struck onto YPD plates buffered at pH 6.0 and incubated at permissive (25°C) or non-permissive (35°C) temperatures. At 35°C, *sec9-7* mutants showed poor growth and failed to form individual colonies, while *sec9-7 vma1Δ* double mutants displayed increased growth and colony formation. The large colonies in *sec9-7* sectors are likely extragenic reverants that are noticeably absent in *sec9-7 vma1Δ* sectors.

The V-ATPase has been implicated in cytosolic pH homeostasis in yeast and *in vitro* experiments have shown that the rate of SNARE complex formation is influenced by pH [35], [36]. While it is difficult to translate *in vitro* assembly results into *in vivo* effects, the improved growth of *sec9-4* mutant cells upon loss of the V-ATPase could be due to changes in SNARE complex formation caused by alterations of intracellular pH. To test this, we assessed whether the pH of the medium affects the growth of *sec9* mutants. Cells with temperature-sensitive mutations in *sec9* were plated on media buffered at different pH and incubated at different temperatures (Figure 3a). Wild-type cells grew and formed individual colonies independent of pH or temperature. In contrast, *sec9-4* mutants showed improved growth at the semi- and non-permissive temperature when grown on medium buffered at pH 8.0 compared to pH 6.0. These results suggest that suppression of *sec9-4* in the absence of the V-ATPase and at alkaline pH could be caused by the same mechanism, possibly the result of altered pH homeostasis.

We tested whether suppression is specific to the *sec9-4* allele by assaying the growth properties of *sec9-7* cells under alkaline conditions and in the absence of the V-ATPase. At 30°C, *sec9-7* cells grew very poorly on media that was buffered to pH 6.0 yet displayed near wild-type growth on pH 8.0 media (Figure 3a). The growth properties of *sec9-7* mutants were also increased in the absence *VMA1*. At 35°C, *sec9-7* mutants showed poor growth and failed to form individual colonies, while *sec9-7 vma1Δ* double mutants grew and formed individual colonies (Figure 3b). These results indicate that suppression is not specific to the *sec9-4* allele. Although suppression is not *sec9* allele specific, it is specific for mutations within *sec9*. Other *sec^ts^* alleles that affect late stages in post-Golgi secretion were analyzed and none of the tested mutations were suppressed by either *vma1Δ* or growth at elevated pH (Table 1). Together these results demonstrate that suppression is specific to mutations in *sec9*, which suggests that suppression reflects a bypass or restoration of a specific function that is impaired when *SEC9* is mutated, rather than general defects in secretion.

**Table 1:** Summary of growth characteristics of *sec^ts^* mutants at elevated pH and in combination with *vma1Δ*.

Allele	Growth at pH 8.0	Growth with *vmaΔ*
*sec1-1*	no effect	no effect
*sec2-41*	no effect	no effect
*sec3-2*	no effect	not determined
*sec4-8*	no effect	no effect
*sec5-24*	no effect	no effect
*sec6-4*	no effect	slightly worse
*sec8-9*	no effect	not determined
*sec10-2*	no effect	not determined
*sec15-1*	worse	not determined
*sec9-4*	better	better
*sec9-7*	better	better

### Increased SNARE complex formation does not result in suppression

**Figure 4:**
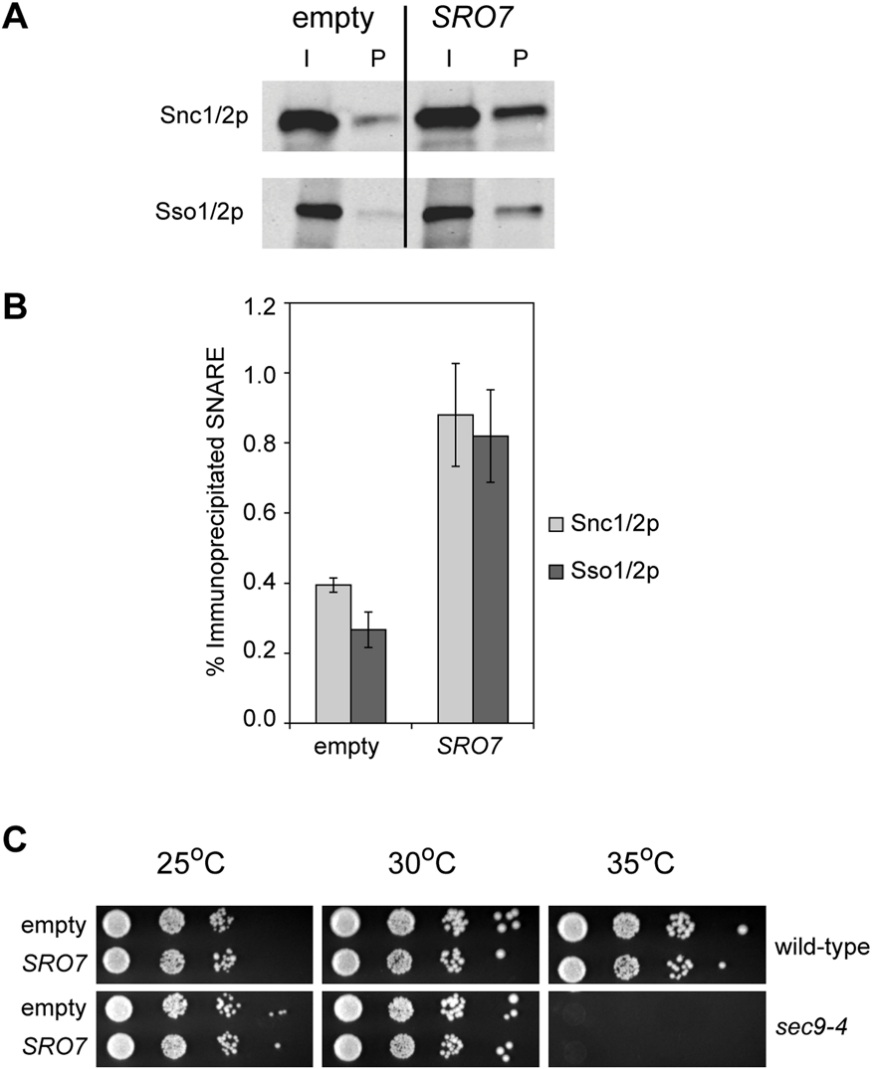
Increased SNARE complex formation does not result in suppression of *sec9-4*. (A) Overexpression of *SRO7* causes an increase in SNARE complex formation. Immunoprecipitates of *HA-SEC9* strains harboring high-copy number *SRO7* or empty vector were probed by western blot with antibodies against Sso1/2p or Snc1/2p. The lanes labeled P are the immunoprecipitation pellets, while the input lanes represent 5% of the lysate from which the immunoprecipitate was derived. (B) Quantification of the immunoprecipitation in A. Integrated intensity of input and pellet bands for each genotype was determined by infrared imaging and used to determine the percent of immunoprecipitated SNARE. There is an increase in the amount of Sso1/2p and Snc1/2p that is immunoprecipitated in strains with *SRO7* overexpression plasmids compared to empty-vector controls (P<0.05, two-tailed T-test, n = 3). Columns represent the mean+/−SEM. Similar levels of HA-Sec9p were immunoprecipitated from both genotypes. (data not shown). (C) Overexpression of *SRO7* does not result in suppression of *sec9-4*. Serial dilutions of wild-type and *sec9-4* cells harboring the indicated overexpression plasmid were spotted on selective media (SC-Ura) and incubated at the indicated temperature for 3 days. The growth characteristics of *sec9-4* cells harboring *SRO7* on an overexpression plasmid were indistinguishable from empty vector controls at all temperatures tested.

One possible mechanism to explain the increased growth of *sec9* mutations in the absence of the V-ATPase or on alkaline media is through an elevation of SNARE complex formation under suppressing conditions. If this were the case, then increasing SNARE complex formation by other means would be expected to also result in suppression of *sec9-4*. To identify conditions that increase SNARE complex formation, we tested whether overproduction of a protein that interacts with Sec9p influences the levels of *in vivo* SNARE complexes. Sro7p is an effector of Sec4p that binds directly to Sec9p, and can assemble into a ternary Sro7p-Sec4p-Sec9p complex [24], [23]. In addition, Sro7p plays a positive role in secretion based on phenotypic analysis of loss-of-function mutants as well as genetic studies demonstrating suppression of a broad range of secretory mutations by *SRO7* overexpression [19]. We assayed SNARE complex formation in strains transformed with high-copy number plasmids containing *SRO7* or empty vector. Increased expression of *SRO7* from a high-copy plasmid did not have any measurable effect on the steady-state levels of Sso1/2p or Snc1/2p (Figure 4 and data not shown). SNARE complex formation was assayed by immunoprecipitation of HA-Sec9p and probing the pellets for Sso1/2p and Snc1/2p by western blot (Figure 4a and b). When compared to empty-vector controls, strains bearing *SRO7* plasmids showed an increase of approximately two-fold in the amount of Snc1/2p and Sso1/2p that co-precipitated with HA-Sec9p. We next asked whether increased SNARE complex formation is associated with suppression by testing *SRO7* overexpression plasmids for suppression of the *sec9-4* growth defect. The growth characteristics of *sec9-4* strains harboring *SRO7 on* a high-copy number plasmid were indistinguishable from empty vector controls at permissive and non-permissive temperatures (Figure 4c). These results demonstrate that increased SNARE complex formation does not result in suppression of *sec9*-4 and therefore suggest that the suppression of *sec9* mutations, by loss of *VMA* function or increased pH of the media, is unlikely the result of increased SNARE assembly.

### Suppressing conditions are not associated with increased SNARE complex formation

**Figure 5:**
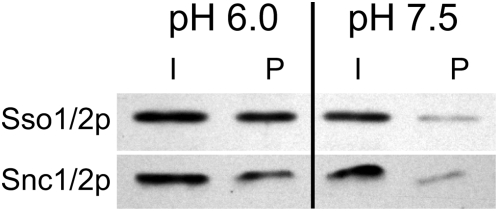
Suppressing conditions are associated with decreased SNARE complex formation. Cells grown under alkaline conditions have reduced levels of SNARE complexes. *HA-SEC9* cells were grown in rich media buffered to pH 6.0 or pH 7.5. Lysates from these cells were prepared and levels of SNARE complex formation assayed by immunoprecipitation with antibodies against HA-Sec9p and subsequent western blotting for Snc1/2p and Sso1/2p. Input lanes (I) represent 2% of the lysate that was immunoprecipitated and loaded in the pellet lanes (P). The relative amount of Sso1/2p and Snc1/2p that is co-immunoprecipitated with HA-Sec9p is reduced in lysates from cells grown at pH 7.5 relative to the amount of precipitated SNAREs from cells grown at pH 6.0.

To directly test whether pH-dependent suppression is due to increased SNARE complex formation, we analyzed *in vivo* SNARE complex formation in cells grown in media buffered at different pH (Figure 5). Strains expressing HA-Sec9p were grown in media that was buffered at low (6.0) or high (7.5) pH. The amount of Sso1/2p and Snc1/2p that was pulled down with Sec9p was dramatically decreased in cells grown at pH 7.5 when compared to cells grown at pH 6.0. This result demonstrates that suppression of *sec9-4* is not due to an increase in SNARE complex formation. We also observed a difference in the growth rates of wild-type cells in liquid media buffered at different pH. At pH 7.5, the growth rate of wild-type cells was much lower than that of cells grown in media buffered at pH 6.0 and no growth was observed in liquid media buffered at pH above 7.5 (data not shown).

### Slow growth conditions suppress the temperature-sensitivity of *sec9* mutations

**Figure 6:**
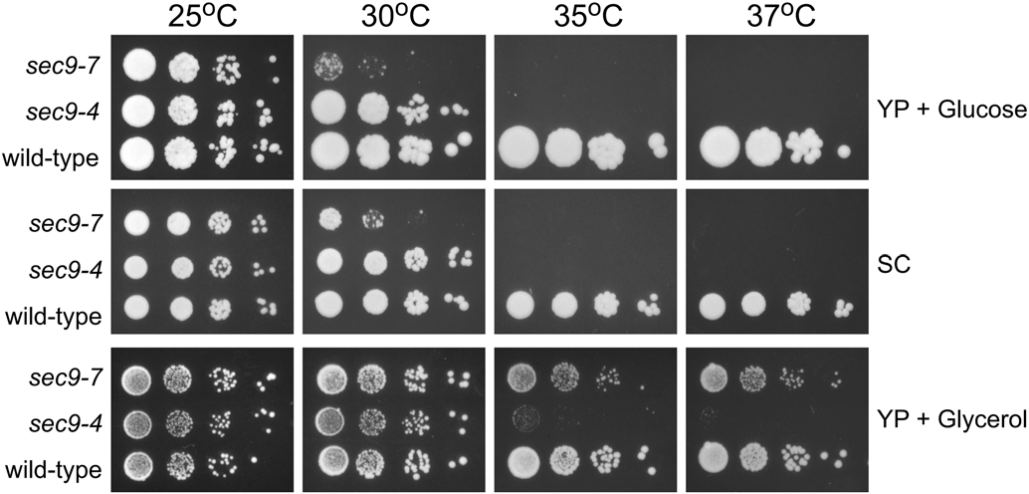
Decreased growth suppresses the temperature-sensitivity of *sec9* alleles. Serial dilutions of *sec9-7*, *sec9-4*, or wild-type cell suspensions were spotted onto rich media containing glucose (YP+glucose), synthetic complete media (SC) or rich media with glycerol (YP+glycerol) and incubated at the indicated temperature for 3 days (YP+glucose and SC) or 5 days (YP+glycerol). On YP+glucose, *sec9* mutants are unable to grow at temperatures above 35°C and show decreased growth at 30°C, relative to wild-type cells. Compared to YP+glucose, the growth of wild-type cells is decreased when plated on SC or YP+glycerol and under these conditions both *sec9-4* and *sec9-7* cells show increased growth at semi- and non-permissive temperatures. The relative increase in fitness associated with growth on YP+glycerol is higher for *sec9-7* mutants compared to *sec9-4* mutant cells.

As noted above, cell growth was inhibited in liquid culture under alkaline conditions. In addition, disruption of V-ATPase subunit genes also caused growth defects when cells were grown on un-buffered media (Figure 1 and data not shown). This led us to hypothesize that the observed suppression of *sec9* might be the result of culture conditions that decrease growth. Slower cell growth would be expected to reduce the rate of secretory vesicle formation and thereby lower the requirement for SNARE function. To test this hypothesis, we asked whether reducing overall growth rates by another means would also result in suppression of *sec9*. Yeast are unable to ferment glycerol, thus the growth rate on media containing glycerol, as the sole carbon source, is much slower than the growth on media that contains glucose. Both *sec9-4* and *sec9-7* mutant cells showed growth defects at 30°C and failed to grow at 35°C or higher on YP+glucose media. In contrast, both cell types grew and formed individual colonies at 35°C and 37°C when plated on YP+glycerol media (Figure 6). There was no difference in the pH of these two types of media. This result demonstrates that the overall growth conditions can influence the phenotype of *sec9* mutants. When compared to wild-type cells, *sec9-7* mutant cells grew better at elevated temperatures than *sec9-4* mutant cells. It was particularly striking that the colony size of *sec9-7* mutants was similar to the size of wild-type colonies at 37°C on YP+glycerol media.

## Discussion

We designed a genetic screen to identify proteins that influence SNARE complex formation by isolating suppressors of mutations defective in SNARE complex formation. Our hypothesis was that *sec9* suppressing conditions (either by extragenic suppressors or changes in culture media) would be due to an increase in SNARE complex formation. Contrary to our hypothesis, we found that suppressing conditions led to a decrease in SNARE complex formation as assayed by co-immunoprecipitation. In addition, we observed suppression of two different alleles of *sec9* that have intrinsic differences in their ability to form SNARE complexes. As an alternative test of whether suppression is linked to increased SNARE complex formation, we examined if genetic enhancement of SNARE complex formation results in suppression of *sec9* mutations. Overexpression of *SRO7* results in increased SNARE complex levels, but does not suppress *sec9* mutations. Together these results exclude the possibility that suppression is due to increased SNARE complex formation.

The results of our screen revealed that disruption of the yeast V-ATPase is able to suppress *sec9* mutations. This was initially intriguing, as V-ATPase subunits physically interact with SNAREs and the V-ATPase has been implicated in membrane fusion in other genetic model systems. In these studies, loss-of-function mutations in V-ATPase subunits produce phenotypes that are attributable to defects in membrane fusion independent of acidification [15]–[17]. In each of these cases, the loss of the V-ATPase results in defects in membrane fusion, suggesting a facilitatory role for the V-ATPase in membrane fusion. However, our results indicate that loss of V-ATPase function suppresses two different *sec9* mutations. This is inconsistent with a facilitatory role for the V-ATPase in secretory vesicle fusion, which predicts that disruption of the V-ATPase would enhance the phenotype of mutations in *sec9*. Although our results establish a genetic interaction between the V-ATPase and SNAREs, this interaction is likely a secondary consequence of the growth defect of V-ATPase subunit mutations.

The results presented here suggest that a function of Sec9p and SNARE complex formation is influenced by the physiological state of the cell. One common feature of the different suppressing conditions is that they all confer a reduction in the growth rate of wild-type cells, while mutant backgrounds that do not affect growth (*pep4Δ prb1Δ*) fail to suppress *sec9* mutations. Our interpretation of these results is that mutations in *SEC9* affect a constitutive function, but that the temperature-sensitive phenotype is a manifestation of greater cellular requirement for this absent function under the increased physiological demands of higher temperature. This is consistent with previous observations that the level of SNARE complexes can be controlled by the availability of Sec9p, suggesting that Sec9p is limiting for SNARE complex formation [37]. Suppressing conditions decrease physiological demands by slowing the growth rate and thereby bring the cells back to normal homeostasis at elevated temperatures. Because Sec9-4p and Sec9-7p have differing propensities to form SNARE complexes, this constitutive function of *SEC9* may be independent of SNARE complex formation. Further characterization of the *sec9* suppression phenotype and molecular genetic studies could be utilized to identify this novel function.

## Materials and Methods

### Yeast Culture Conditions

Standard techniques and media were used for cell growth, strain construction, and transformation [38], [39]. Buffered media was prepared by the addition of 50 mM MES, 50 mM MOPS and adjusting to the indicated pH.

### Isolation and identification of suppressor mutations

Cells harboring the *sec9-4* allele were plated on YPD media and incubated at 30°C. Colonies that formed were selected and back-crossed with wild-type cells and the resulting diploids were subject to meiotic analysis to verify that the suppressing mutations were extragenic and characterization of secondary phenotypes associated with the suppressing mutations, in this case cold-sensitivity and poor growth on media containing glycerol. Once a selectable secondary phenotype was determined, cells containing the suppressor mutation were transformed with a genomic library in YCp50 and transformants were screened for rescue of the secondary phenotype. Plasmids conferring rescue were recovered from yeast and analyzed by restriction mapping and sequencing.

### Yeast Molecular Genetics

Deletion of *VMA1* and *VMA16* was by PCR-mediated gene disruption [40]. For disruption of *VMA1* a *HIS3* deletion cassette was generated by PCR using (ATTCTTAGAGTTAAAAAGCAAATAGAGAAGAAAAGAAACACGGATCCCCGGGTTAATTAA) and (CATCTAACAAATATACCAGAAGATAAATGCTACATATATCGAATTCGAGCTCGTTTAAAC) while the deletion cassette for *VMA16* was generated with (GGAAGGCGAATAAAATACAGGAGCTAGAGCGTGTAAGATACGGATCCCCGGCTTAATTAA) and (TAGCTCGTAAAAACGGAAAAGAAAAGCCTGGTTTGAGCGCGAATTCGAGCTCGTTTAAAC) using pFA6-*HIS5MX* as template.

HA-Sec9p was generated by transformation of a *SEC9::HIS3:P_GAL1_-HA*_3_ *-SEC9* cassette into diploid *MATa/MATα his3-Δ200/his3-Δ200 leu2-3,112/leu2-3,112 ura3-52/ura3-52* cells. The transformation amplicon was generated by PCR using (TTGATCCTGCTTTTAACAATTTCGAATCGTTTGCGTAATTGAATTCGAGCTCGTTTA AAC) and (CCTCCTCTGGAGGCTTAATCTTAAAAAATTTCTTTAATCCGCACTGACGAGCGTAATCTG) using pFA6a-*HIS3MX6*-*P_GAL1_*-*HA_3_* as a template, resulting in a transformation cassette directed in-frame to the N-terminus of Sec9p. Diploid transformants were sporulated and subjected to meiotic segregation analysis. Tetrads exhibited a 2∶2 segregation pattern for histidine prototrophy that was linked with the inability to grow on YPD media. Histidine prototrophic spores were tested for production of HA-Sec9p by western blotting using antibodies against HA.

*SRO7-URA3-2*μ was generated by amplifying *SRO7* using (GGACTAGTCCTGAAGCTAATCCTTAACAGCGG) and (CGGGATCCACGTCTCAAAACAATTGGGCC) on a yeast genomic DNA template with Expand High-Fidelity polymerase, digesting with BamHI and SpeI, and ligation into the BamHI-SpeI sites of pRS426. This construct was verified by restriction digest.

### *In vivo* SNARE complex formation assays

Immunoprecipitation experiments were performed as previously described [41], [42] with minor modifications. Cells inoculated from saturated cultures were grown overnight in selective media at 25°C to an OD600 of ∼0.4–1.0. Cells were harvested and washed in ice-cold TAF buffer (20 mM Tris-Cl pH 7.5, 20 mM NaN3, 20 mM NaF) and suspended in ice-cold IP buffer (50 mM HEPES, pH 7.4, 150 mM KCl, 1 mM EDTA, 1 mM DTT, 0.5% NP-40, 2× Complete protease inhibitor (Roche)). Crude cell lysates were generated by beating with 0.5 mm zirconia/silica beads for 2 cycles of 4 minutes each in a Mini-beadbeater 8 at 4°C (Biospec). Lysates were cleared by centrifugation for 20 minutes at 15,000 rpm in a microcentrifuge and the supernatants were assayed for protein concentration by Bradford assay using BSA as a standard. Cleared lysates were diluted to an equal protein concentration and equal volume aliquots were then pre-cleared with ∼80 ul of equilibrated IgG-Sepharose beads by rocking samples for 30 minutes at 4°C, spinning down the beads (30 s at 1000×g) and keeping the supernatant. Epitopes were bound by adding 10 µl of anti-HA antibody (Covance) and incubated with rocking overnight at 4°C. Immuno-complexes were precipitated by the addition of ∼20 µl IgG-Sepharose beads and incubation for 2 hours, followed by spinning the beads down (1000×g, 30 s) and washing 5 times with ice-cold IP buffer. Proteins were elutated from the beads by boiling in 1× SDS-sample buffer. Precipitates and input samples were subject to SDS-PAGE, transferring to nitrocellulose membranes and Western blotting with antibodies against Sso1/2p or Snc1/2p. Assay of percent SNARE immunoprecipitation (Figure 4b) was determined using the Odyssey Infrared Imaging System and comparing the integrated intensity of bands in pellet lanes to bands present in samples derived from 5% of the lysate used for immunoprecipitation.
